# Horizontal transmission of *Salmonella Typhimurium* among German cockroaches and its possible mechanisms

**DOI:** 10.1002/ece3.10070

**Published:** 2023-05-08

**Authors:** Madison Mond, Jose E. Pietri

**Affiliations:** ^1^ Division of Basic Biomedical Sciences, Sanford School of Medicine University of South Dakota Vermillion South Dakota USA

**Keywords:** cockroach, coprophagy, emetophagy, necrophagy, *Salmonella*, transmission

## Abstract

German cockroaches (*Blattella germanica*) can be both mechanical and biological (amplifying) vectors of enteric pathogens, including *Salmonella enterica* serovar Typhimurium (*S. Typhimurium*), which they acquire by feeding upon contaminated substances. *Blattella germanica* is also a gregarious species that shelters in groups and partakes in unique feeding behaviors such as conspecific coprophagy, necrophagy, and emetophagy. These properties create an interphase for potential horizontal transmission of pathogens among cockroach populations through the fecal‐oral route, which could in turn enhance transmission to humans and other animals. Here, we performed a series of experiments to determine: (1) whether horizontal transmission of *S. Typhimurium* infection takes place in *B. germanica*, (2) the prevalence of the phenomenon, and (3) the route(s) through which it may occur. We reveal that true horizontal transmission of *S. Typhimurium* occurs among *B. germanica.* That is, uninfected cockroaches acquire infection of the gut when co‐housed with orally infected conspecifics, albeit at low frequency. Furthermore, we provide definitive evidence that coprophagy and necrophagy are routes of transmission but could not exclude sharing of food or water as contributing routes. On the contrary, transmission by emetophagy appears less likely as oral regurgitates from infected cockroaches contained *S. Typhimurium* for less than one day after ingesting the bacteria. Together, our data enhance current understanding of the ecology of vector‐borne *S. Typhimurium* transmission by cockroaches, implicating conspecific horizontal transmission as a phenomenon that contributes to maintaining infected cockroach populations independently of contact with primary sources of the pathogen. Although the relative importance of horizontal transmission of pathogens in cockroaches in the field remains to be determined, these results also highlight the important role that food and water sources in the local environment may play in cockroach‐borne pathogen transmission and emphasize the importance of sanitation for not only abating infestations but also mitigating pathogen transmission.

## Graphical abstract

We reveal that true horizontal transmission of *Salmonella Typhimurium* occurs among *Blattella germanica*. That is, uninfected cockroaches acquire infection of the gut when co‐housed with orally infected conspecifics, albeit at low frequency. Furthermore, we provide definitive evidence that coprophagy and necrophagy are routes of transmission but could not fully exclude sharing of food or water as contributing routes. These data enhance current understanding of the ecology of vector‐borne *S. Typhimurium* transmission by cockroaches, implicating conspecific horizontal transmission as a mechanism that contributes to maintaining infected cockroach populations independently of contact with primary sources of the pathogen.

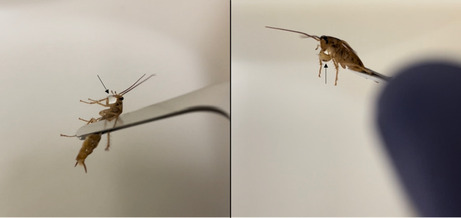

## 1 INTRODUCTION

German cockroaches (*Blattella germanica*) have been epidemiologically and experimentally implicated as vectors of *Salmonella* spp., including *Salmonella enterica* serovar Typhimurium (*S. Typhimurium*), a widespread causative agent of human gastroenteritis (Ash & Greenberg, 1980; Graffar & Mertens, 1950; Kopanic et al., 1994; Nasirian, 2019; Turner et al., 2022). Transmission of pathogenic bacteria such as *S. Typhimurium* by cockroaches can occur through both physical transfer on the cuticle as well as ingestion and subsequent excretion in the feces. Importantly, although transfer on the cuticle is a passive mechanical mechanism of transmission, ingestion of *S. Typhimurium* by *B. germanica* results in active colonization of the gut. In this environment, the bacteria replicate and can persist for weeks while also being disseminated in the feces (Ash & Greenberg, 1980; Turner et al., 2022).

*Blattella germanica* is a gregarious species, and its behavioral ecology is unique among insect vectors in several ways. In both the laboratory and field, the insects have a strong propensity to aggregate within shelters in groups of all life stages. This behavior is driven in part by bacteria‐derived, aggregation‐inducing compounds present in the feces (Wada‐Katsumata et al., 2015) and provides benefits for growth and reproduction (Gemeno Marín et al., 2011; Kopanic et al., 2001). German cockroaches are also omnivores that can subsist on a wide range of diets and adapt to locally available foods (Kells et al., 1999; McPherson et al., 2021; Raubenheimer & Jones, 2006). At night, German cockroaches depart from shelters and forage for food and water sources. They first and most frequently utilize resources closest to harborage sites (Rivault & Cloarec, 1991; Silverman, 1986). They appear capable of associating locations with resources and thus may repeatedly visit sources of food and water (Durier & Rivault, 2000a, 2001). Moreover, *B. germanica* frequently grooms its appendages and partakes in conspecific coprophagy (Durier & Rivault, 2000b; Kopanic et al., 2001; Kopanic & Schal, 1997, 1999; Silverman et al., 1991), necrophagy (Gahlhoff et al., 1999; Tabaru et al., 2003), and emetophagy (Buczkowski et al., 2008; Buczkowski & Schal, 2001).

The aforementioned factors together create ideal circumstances for the horizontal transmission of microbes, including human pathogens, within cockroach populations through the fecal‐oral route. That is, foraging individuals that become infected with *S. Typhimurium* by feeding or drinking from a contaminated source have a high potential to spread the bacteria to uninfected individuals sharing the same shelter and foraging area. As the bacteria replicate in the gut and are shed in the feces from 1 to 20 days after ingestion (Ash & Greenberg, 1980; Turner et al., 2022), infectious feces are likely to be deposited at harborage sites by foraging insects. Coprophagy by uninfected insects could then result in new infections. Oral excretions such as regurgitate, which have been shown to be attractive to conspecifics (Buczkowski & Schal, 2001), could similarly contain infectious bacteria and their consumption by uninfected insects (emetophagy) could be an additional route of transmission. Furthermore, some species of *Salmonella* may survive in cockroach cadavers for up to 10 days (Singh et al., 1995) and thus may be transmissible through necrophagy of infected insects. Lastly, shared sources of food and water near harborage sites may become contaminated by infected cockroaches and serve as means of spread to other foraging individuals. Such forms of horizontal transmission could have significant implications for public health by increasing and/or maintaining populations of infected cockroaches at a locale independently of contact with a primary pathogen source, especially when considering bacterial amplification within the cockroach gut (Turner et al., 2022).

A previous study preliminarily examined horizontal transmission of *S. Typhimurium* in the American cockroach (*Periplaneta americana*), a species in the same order but different family than *B. germanica* (Kopanic et al., 1994). This study revealed that American cockroaches infected orally with *S. Typhimurium* could contaminate uninfected individuals when co‐housed. Nonetheless, the experimental design of Kopanic et al. did not distinguish between external contamination of the cockroaches and true infection (i.e., of the gut), did not enable identification of the route(s) of horizontal transmission, and excluded *B. germanica* from horizontal transmission experiments. Given the potential importance of horizontal transmission of human pathogens among *B. germanica*, which is one of two cockroach species that completes its lifecycle entirely indoors, here we designed a series of experiments to determine: (1) whether true horizontal transmission of *S. Typhimurium* infection takes place in this species, (2) the prevalence of the phenomenon, and (3) the route(s) through which it may occur.

## 2 MATERIALS AND METHODS

### 2.1 Cockroach colonies

The American Cyanamid Orlando laboratory strain of *B. germanica* was used in the present study. Cockroach colonies were maintained in plastic enclosures at 25 ± 1°C and 40%–45% relative humidity on a 12:12 (L:D) h photoperiod. The colonies had free access to dog chow (Purina, St. Louis, MO), tap water, and egg carton harborages. Adult males were primarily used in experiments in order to preserve females for colony propagation and minimize physiological variation due to gonadotropic and developmental cycles.

### 2.2 Salmonella culture

The well‐characterized 14028s strain of *S. enterica* serovar Typhimurium (*S. Typhimurium*) expressing GFP from a plasmid (pDW5) was used in this study. This strain encodes both green fluorescence and kanamycin resistance markers. Cultures were grown on a shaker in liquid LB medium (Sigma Aldrich, St. Louis, MO) at 37°C. Prior to each experiment, the viability of the culture used was confirmed by plating on selective LB agar plates containing 100 μg/mL kanamycin.

### 2.3 Infection of donor cockroaches with *Salmonella*

**FIGURE 1:**
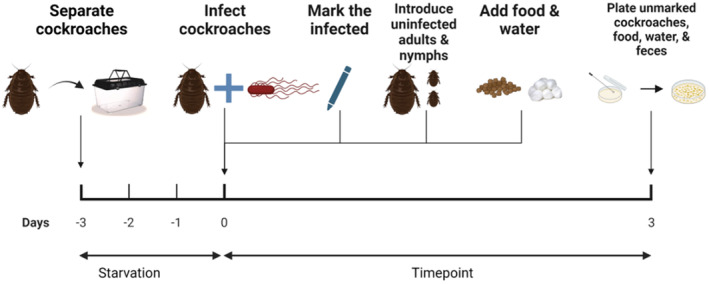
Experimental design. Groups of marked cockroaches were orally infected with *Salmonella Typhimurium* to serve as donors. Infected donors were then co‐housed with unmarked adult males and/or early instar nymphs that served as uninfected recipients. Uninfected recipient insects were examined for internal *S. Typhimurium* infection via culture three days after co‐housing with infected donors. Food sources, water sources, and feces at harborage sites were also tested for *S. Typhimurium* via culture. Figure was created in BioRender (license YR252GG6Q3).

Groups of donor cockroaches were marked with color tags and orally infected with *S. Typhimurium* as described in our previous studies (Turner et al., 2022; Turner & Pietri, 2022). In brief, cockroaches were separated into experimental enclosures and starved of food and water for three days, which is sufficient to promote consistent feeding among individuals without causing mortality. Then, a shallow Petri dish containing a stationary‐phase culture of *S. Typhimurium* diluted to OD_600_ = 1 was provided to the cockroaches as a sole food source for 30 min. This concentration results in an average ingested bacterial load of ~3.56 × 10^6^ CFU per insect (Turner et al., 2022), which is a dose that could be acquired through consumption of several milligrams of infected human or pig feces in the field (Buchwald & Blaser, 1984; Tanaka et al., 2014). Nontoxic blue food dye (blue #1) was added to the cultures to enable tracking of fed cockroaches (Martins et al., 2018). Insects that did not feed on the bacteria during the 30‐min period were removed and not used for experiments. The infected donor roaches were then co‐housed with uninfected recipient cockroaches for three days in 5 in. × 8 in. plastic arenas. Co‐housing took place under four different experimental conditions as follows, with each being identically replicated three or four times. The experimental design is outlined in Figure 1.

#### 2.3.1 Experiment 1

In Experiment 1, 10–15 infected male cockroaches served as donors and were co‐housed with 10–15 uninfected recipient adult males and 10–15 recipient early instar nymphs. The recipient cockroaches were not starved prior to co‐housing. Solid dog chow pellets were present as a food source and a cotton ball soaked in tap water was present as a water source.

#### 2.3.2 Experiment 2

In Experiment 2, 10–15 infected male cockroaches served as donors and were co‐housed with 10–15 uninfected recipient adult males and 10–15 recipient early instar nymphs. The recipient cockroaches were not starved prior to co‐housing. No food or water sources were present in the arenas.

#### 2.3.3 Experiment 3

In Experiment 3, 10–15 infected male cockroaches served as donors and were co‐housed with 10–15 uninfected recipient adult males and 10–15 recipient early instar nymphs. The recipient cockroaches were not starved prior to co‐housing. Solid dog chow pellets were present as a food source. However, instead of tap water, a cotton ball soaked in liquid LB medium was present as a water source. In contrast to tap water, this readily consumed medium supports the growth of any bacteria deposited by infected donors.

#### 2.3.4 Experiment 4

In Experiment 4, 4–5 infected early instar nymphs served as donors. Immediately after infection, these insects were cold anesthetized and killed by crushing the head with forceps. The infected nymph cadavers were then provided to 10 uninfected recipient adult males as a sole food source. The recipient cockroaches were not starved prior to co‐housing. No other sources of food or water were present in the arenas.

### 2.4 Culture of *S. Typhimurium* from recipient cockroaches

Following the three‐day experimental co‐housing period, a subset of the recipient cockroaches were collected and examined for horizontally acquired internal *S. Typhimurium* infection via selective culture. Critically, individual insects were surface sterilized to remove contaminating bacteria from the cuticle surface by successive washes in 10% bleach, 70% ethanol, and deionized water. Then, the insects were homogenized in sterile PBS using a handheld tissue homogenizer (Omni International, Kennesaw, GA). Insect homogenates were diluted in PBS, spread on selective LB agar plates containing 100 μg/mL kanamycin, and incubated overnight at 37°C to detect *S. Typhimurium* colonies. The calculated limit of detection (LOD) of this plating assay was 50 bacteria/cockroach and insects were scored as positive or negative for infection. In each experiment, working stocks of PBS were plated as negative controls to account for contamination and these showed no growth on kanamycin plates. Furthermore, uninfected cockroaches from our colonies maintained identically were plated on LB kanamycin and showed no growth of *S. Typhimurium*.

### 2.5 Culture of *S. Typhimurium* from food, water, and feces at harborage sites

In each experiment, we also tested the food and water sources present in the area as well as samples of feces deposited in harborages for the presence of *S. Typhimurium* via culture. Dog chow pellets and cotton balls containing water or LB were soaked in PBS for 30 min and the solutions were directly plated on selective LB agar containing 100 μg/mL kanamycin. Feces was collected from enclosures using a sterile swab soaked in LB medium, and this solution was also directly spread on LB kanamycin plates. Lastly, infected donor nymph cadavers were pooled, homogenized in sterile PBS, and the homogenates were cultured as described above for recipient cockroaches. Each sample was scored as positive or negative for *S. Typhimurium*.

### 2.6 Culture of *S. Typhimurium* from oral regurgitates of donor cockroaches

**FIGURE 2:**
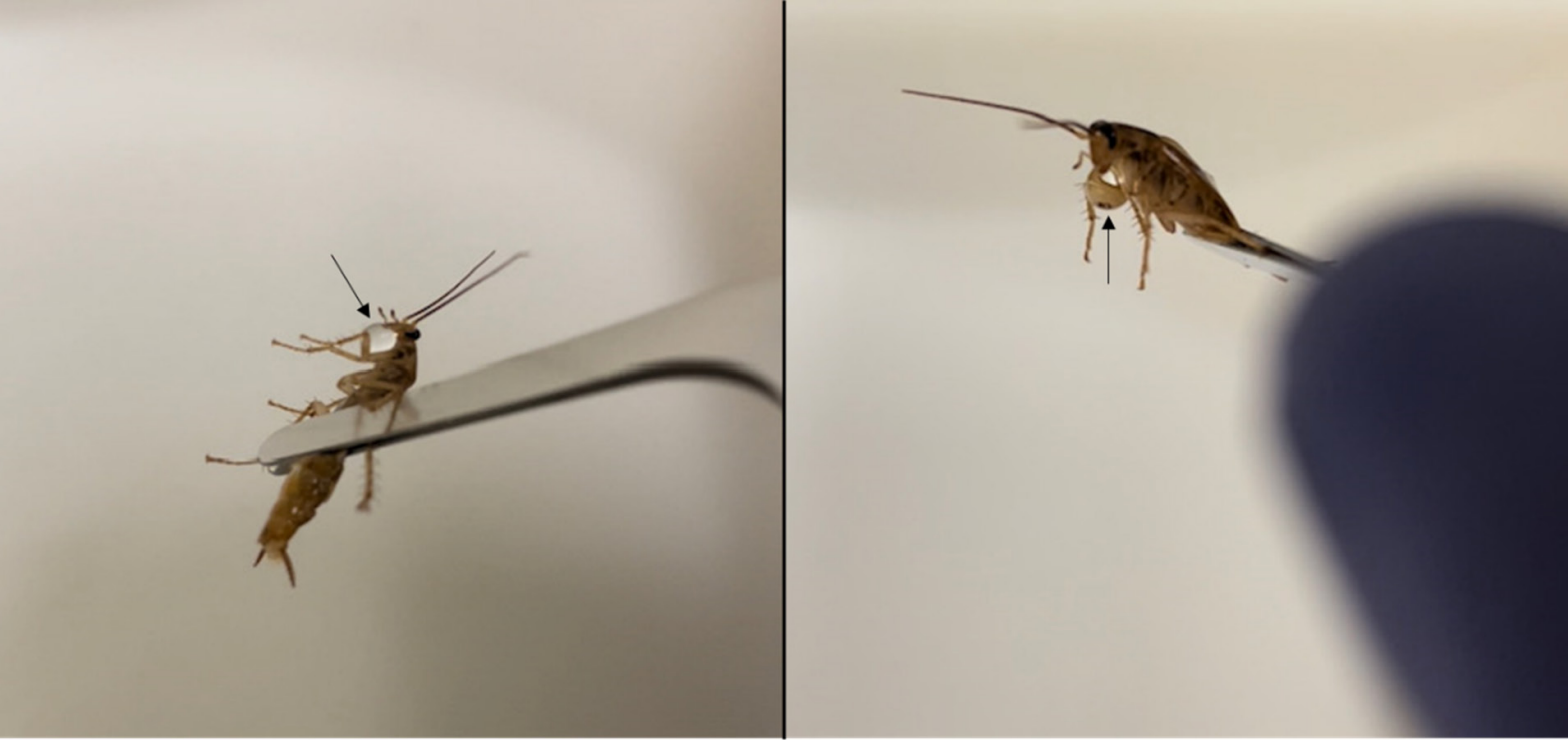
Regurgitation of oral excretions by infected cockroaches upon physical disruption. Cockroaches were induced to regurgitate oral excretions (arrows) by gently grasping them with soft forceps immediately after feeding on a *Salmonella Typhimurium* culture, or 24 or 72‐h later (shown in images). The resulting regurgitates were directly cultured to detect *S. Typhimurium*.

In separate experiments, we tested whether oral regurgitates produced by infected cockroaches contained viable *S. Typhimurium*. Regurgitation of oral excretions, in addition to being a symptom of insecticide toxicity (Buczkowski & Schal, 2001), can also be triggered by physical disruption and exposure to temperature extremes. In our experiments, when cockroaches were probed or gently grasped with soft forceps, they rapidly regurgitated small liquid droplets (Figure 2). We induced infected adult males to produce these regurgitates immediately after feeding on *S. Typhimurium* cultures or 24‐ or 72 h later by gently grasping them with soft forceps. The regurgitated droplets were directly spotted onto selective LB agar plates containing 100 μg/mL kanamycin and incubated overnight at 37°C to detect *S. Typhimurium* colonies.

## 3 RESULTS

### 3.1 Horizontal transmission of infection in enclosures with food and tap water as a water source

When infected donor cockroaches were co‐housed with uninfected recipients in the presence of dog chow pellets and tap water (Table 1), horizontal transmission of *S. Typhimurium* was detected, albeit at a low rate. Across four independent replicates, a total of seven of 30 adult males (23.3%) horizontally acquired *S. Typhimurium* internally. On the contrary, horizontal acquisition of infection was not detected in 21 nymphs tested. In this set of experiments, feces deposited by donor cockroaches at the harborage site were always positive for *S. Typhimurium*. Meanwhile, the water source was positive in two of four replicates and the food (dog chow pellet) in only one of four replicates. In one replicate (replicate 3), horizontal transmission to adult males occurred in the absence of contaminated food or water. Together, these results provided evidence of true horizontal transmission but could not discern the route(s) of transmission.

**TABLE 1:** Horizontal transmission of infection in enclosures with dog chow pellets and tap water present.

Replicate	# of recipient adults infected/adults tested	# of recipient nymphs infected/nymphs tested	Food	Water	Feces in harborage
1	0/7	0/7	−	+	+
2	0/8	0/8	−	−	+
3	3/7	0/7	−	−	+
4	4/8	Not tested	+	+	+

*Note*: “+” indicates *Salmonella Typhimurium* was detected by culture. “−” indicates *Salmonella Typhimurium* was not detected.

### 3.2 Horizontal transmission of infection in the absence of food or water

Because we detected horizontal transmission of *S. Typhimurium* in the absence of contaminated food or water, we next sought to determine whether horizontal transmission could take place via coprophagy. To do so, we examined horizontal transmission in the absence of any food or water sources (Table 2). In these experiments, the feces deposited at the harborage site by infected donors were again always positive for *S. Typhimurium*. Furthermore, horizontal transmission to adult male recipients was detected in three of three replicates. In total, six of 21 (28.6%) adult male recipients horizontally acquired internal *S. Typhimurium* when co‐housed with infected donors in the absence of food and water. Horizontal acquisition of infection was not detected in 21 recipient nymphs tested. Nonetheless, the acquisition of internal *S. Typhimurium* from infected donors by some recipient cockroaches in the absence of any shared food or water sources definitively implicated coprophagy as a route of horizontal transmission.

**TABLE 2:** Horizontal transmission of infection in enclosures lacking food or water sources.

Replicate	# of recipient adults infected/adults tested	# of recipient nymphs infected/nymphs tested	Feces in harborage
1	2/7	0/7	+
2	1/7	0/7	+
3	3/7	0/7	+

*Note*: “+” indicates *Salmonella Typhimurium* was detected by culture. “−” indicates *Salmonella Typhimurium* was not detected.

### 3.3 Horizontal transmission of infection in enclosures with food and LB medium as a water source

To further examine the possible impact of different environmental food and water sources on horizontal transmission, we tested horizontal transmission in the presence of a nutritive liquid medium (LB medium) as a water source in addition to dog chow pellets (Table 3). In contrast to tap water, LB medium enables the growth of any *S. Typhimurium* deposited by foraging infected donors. In this set of experiments, the feces deposited at the harborage site by infected donors were always positive for *S. Typhimurium*. The solid food was positive in two of three replicates, and the liquid medium was positive in three of three replicates. Interestingly, however, horizontal acquisition of *S. Typhimurium* was documented in only one of 21 adult males and one of 21 nymphs (4.76%) across three replicates, suggesting that the composition of food/water sources in the environment may influence environmental dissemination of pathogens by infected cockroaches as well as horizontal transmission rate.

**TABLE 3:** Horizontal transmission of infection in enclosures with dog chow pellets and LB medium as a water source.

Replicate	# of recipient adults infected/adults tested	# of recipient nymphs infected/nymphs tested	Food	LB	Feces in harborage
1	0/7	0/7	+	+	+
2	1/7	0/7	+	+	+
3	0/7	1/7	−	+	+

*Note*: “+” indicates *Salmonella Typhimurium* was detected by culture. “−” indicates *Salmonella Typhimurium* was not detected.

### 3.4 Horizontal transmission of infection via consumption of infected cadavers

We additionally tested the role of necrophagy in horizontal transmission of *S. Typhimurium* by co‐housing uninfected adult male recipients with cadavers of infected donor nymphs in the absence of any other food or water sources (Table 4). In three of three replicates, infected donor cadavers were partially consumed by recipient adults and the remaining bodies harbored viable *S. Typhimurium* for the duration of the experiment, indicating the bacteria survive in cadavers for at least 3 days. Moreover, in two of three replicates, horizontal acquisition of *S. Typhimurium* by necrophagy was detected. In total, *S. Typhimurium* was detected in five of 21 (23.8%) of recipient adults tested.

**TABLE 4:** Horizontal transmission of infection in enclosures containing no water source and infected nymph cadavers as a sole food source.

Replicate	# of recipient adults infected/adults tested	Nymph cadavers
1	3/7	+
2	0/7	+
3	2/7	+

*Note*: “+” indicates *Salmonella Typhimurium* was detected by culture. “−” indicates *Salmonella Typhimurium* was not detected.

### 3.5 Regurgitation of *Salmonella* by infected cockroaches

We lastly explored the potential involvement of emetophagy in horizontal transmission of *S. Typhimurium*. Oral regurgitates induced from cockroaches immediately after feeding on a *S. Typhimurium* culture were blue in color, indicating that they contained material from the foregut, including freshly ingested bacteria. As expected, when these regurgitates were cultured on selective agar plates containing kanamycin, they were always positive for *S. Typhimurium* (6/6 insects). However, regurgitates induced 24‐ or 72‐h postinfection were not blue (Figure 2) and when these were cultured, they were all negative for *S. Typhimurium* (6 insects per time point). Similarly, regurgitates from uninfected control cockroaches were always negative (6/6).

## 4 DISCUSSION

The results presented here demonstrate that true horizontal transmission of *S. Typhimurium*, and not just external contamination, can take place among German cockroaches. That is, orally infected cockroaches can disseminate the bacteria at harborage sites and in foraging areas in multiple ways. These disseminated bacteria can then be ingested by uninfected cockroaches and infect the gut.

One definitive route of horizontal transmission is coprophagy. We first observed horizontal transmission in an experiment in which food and water were available in the arena but had not been contaminated, suggesting the involvement of coprophagy (Table 1). Indeed, when experiments were conducted in which no food or water was available besides the feces produced by infected donors, horizontal transmission still occurred (Table 2). This result implicated coprophagy definitively. As such, we have established a new role for coprophagy in transmission of human pathogenic microbes by *B. germanica*. Coprophagy is known to be important for acquisition of the commensal microbiota (Kakumanu et al., 2018; Rosas et al., 2018) and other aspects of physiology, and our data now show that the behavior also contributes to the vector potential of cockroaches. All life stages of *B. germanica* partake in coprophagy, but the behavior is most pronounced in early nymphs (Silverman et al., 1991). Yet, in our experiments, mostly adults and not nymphs horizontally acquired *S. Typhimurium* from infected donors. While this is somewhat unexpected if transmission is occurring via coprophagy, we have previously shown that nymphs are more resistant to ingested *Escherichia coli* than adults (Ray et al., 2020), and the same may be true for *S. Typhimurium*, offering a possible explanation for the difference in horizontal transmission to nymphs vs adults that should be further investigated.

In several experiments where food or water sources were contaminated, horizontal transmission did not happen (e.g., experiment 1 replicate 1 and experiment 3 replicate 1), further supporting the primary role of coprophagy. We posit that the primary driver of successful horizontal transmission is the concentration of bacteria ingested. Feces from infected insects contain a high concentration of bacteria (>2000 bacteria excreted/insect/24 h) (Turner et al., 2022). However, cockroaches may not always consume feces present in the enclosure or consume enough of it to become infected, especially if other preferred food sources are present. Yet, food sources, though they may become contaminated, may not necessarily harbor sufficient concentrations of bacteria to infect other cockroaches. Thus, coprophagy could also explain the variation seen in our results and the low overall levels of horizontal transmission.

When the water source in experimental arenas was switched from tap water, which does not enable bacterial growth, to a nutrient‐rich liquid medium (LB) that also provided a food source in addition to dog chow (Table 3), this medium always became contaminated with *S. Typhimurium*, in contrast to water. Nonetheless, horizontal transmission among cockroaches did not increase and appeared to even decrease. This was again consistent with coprophagy as the primary mechanism of transmission, as cockroaches may have been partaking in minimal coprophagy with multiple nutrient sources available. Interestingly, however, this result underscores the role that different sources of food and water in the environment could play in the environmental dissemination of the bacteria by cockroaches. If liquid or nutrient rich food/water sources are present, these are more likely to support bacterial growth when contaminated by cockroaches and may further contribute to the spread of pathogen in the environment.

It is difficult to fully discern the contributions of sharing of food and water to horizontal transmission, and to separate these from the contribution of coprophagy. Our experimental design definitively implicated coprophagy by demonstrating horizontal transmission in the absence of food and water sources, but in initial experiments that included food and water, horizontal transmission may have occurred through coprophagy, contaminated food or water, or a combination of these routes. Nonetheless, our data strongly indicate that solid foods and water may not support transmission well, as these often did not become contaminated by infected cockroaches. In previous studies, infected *P. americana* did not contaminate solid food pellets when feeding on them and infected *B. germanica* contaminated water sources less efficiently than *P. americana* (Kopanic et al., 1994). Our results are consistent with these findings.

In addition to coprophagy, our data also demonstrate that necrophagy is a definitive route of horizontal transmission (Table 4). *S. Typhimurium* remains viable in infected cadavers for at least 3 days and when infected cadavers are a sole food source for uninfected adults, necrophagy results in an appreciable level of horizontal transmission. However, some studies have indicated that necrophagy is a last resort for starving *B. germanica* (Tabaru et al., 2003). Therefore, while also a possible means of transmission, this route may be uncommon in nature.

Emetophagy likely plays a minimal, if any, role in horizontal transmission. Although oral regurgitates produced by infected cockroaches immediately after ingesting *S. Typhimurium* contained viable bacteria, regurgitates produced 24‐ and 72‐h postinfection did not. This finding suggests that either there are minimal viable bacteria present in the foregut after 24 h or that the bacteria that are present are tissue associated and therefore not subject to regurgitation. If oral regurgitates from recently infected cockroaches are attractive, as insecticide induced regurgitates are (Buczkowski et al., 2008; Buczkowski & Schal, 2001), then they could serve as a source of infection for insects that feed upon them. However, this route would only be relevant for a short period of time. It is also unclear whether regurgitation of bacteria by infected cockroaches occurs in the field in the absence of physical stimulation, except perhaps in response to a precisely timed insecticide exposure. Ultimately, the involvement of emetophagy remains difficult to test directly even in the laboratory as the regurgitates must be artificially induced and they desiccate rapidly upon release.

Horizontal transmission is a phenomenon that could potentially enhance cockroach‐borne transmission of infectious agents to humans and livestock. In particular, horizontal transmission could maintain or increase populations of infected cockroaches at a locale independently of contact with primary pathogen sources, especially when considering replication by horizontally acquired *S. Typhimurium* in the gut (Turner et al., 2022). The horizontal transmission process may also help bridge pathogen spread by cockroaches across different environments (e.g., agricultural and urban) (Zurek & Ghosh, 2014). Although we documented relatively low rates of horizontal transmission, our experiments lasted only three days and it is possible that longer periods of contact between infected and uninfected cockroaches could increase transmission rates. Additionally, the ratio of infected donors to uninfected recipients likely influences the dynamics of horizontal transmission and should be further explored. The relative importance of horizontal transmission of pathogens among cockroaches in the field also remains to be determined. Even so, our findings underscore the importance of sanitation and removal of environmental food sources not only for cockroach control (Ko et al., 2016; Schal, 1988; Wang et al., 2019) but also control of pathogen dissemination by these insects.

## AUTHOR CONTRIBUTIONS

**Madison Mond:** Conceptualization (equal); data curation (equal); formal analysis (equal); investigation (equal); methodology (equal). **Jose E. Pietri:** Conceptualization (equal); data curation (equal); formal analysis (equal); funding acquisition (equal); investigation (equal); methodology (equal); project administration (equal); supervision (equal); writing – original draft (equal); writing – review and editing (equal).

## FUNDING INFORMATION

This study was funded by the National Institutes of Health, National Institute of Allergy and Infectious Diseases grant R01AI171014 to JEP. The funder played no role in the study design and execution, nor in the decision to publish.

## CONFLICT OF INTEREST STATEMENT

The authors have no competing interests to declare.

## Data Availability

All data are presented in the manuscript tables.
